# Supplementary material for: Role and mechanism of NCAPD3 in promoting malignant behaviors in gastric cancer
Source: Front Pharmacol. 2024 Apr 22;15:1341039. doi: 10.3389/fphar.2024.1341039 (PMC11070777; doi:10.3389/fphar.2024.1341039)
Supplement: Supplementary file 11 [file DataSheet2.ZIP › GSEA/Canonical pathways/my_analysis.Gsea.1599462267220/REACTOME_VESICLE_MEDIATED_TRANSPORT.html]

Details for gene set REACTOME\_VESICLE\_MEDIATED\_TRANSPORT[GSEA]

|  || Dataset | filtered\_dataset.sample\_info.cls#WT\_versus\_NCAPD3\_MUT |
| Phenotype | sample\_info.cls#WT\_versus\_NCAPD3\_MUT |
| Upregulated in class | NCAPD3\_MUT |
| GeneSet | REACTOME\_VESICLE\_MEDIATED\_TRANSPORT |
| Enrichment Score (ES) | -0.28795695 |
| Normalized Enrichment Score (NES) | -1.7039412 |
| Nominal p-value | 0.016393442 |
| FDR q-value | 0.0900252 |
| FWER p-Value | 0.552 |
Table: GSEA Results Summary

  

Fig 1: Enrichment plot: REACTOME\_VESICLE\_MEDIATED\_TRANSPORT      
 Profile of the Running ES Score & Positions of GeneSet Members on the Rank Ordered List

  

| SYMBOL | TITLE | RANK IN GENE LIST | RANK METRIC SCORE | RUNNING ES | CORE ENRICHMENT || 1 | 160518 | DENND5B | 13 | 1.060 | 0.0358 | No |
| 2 | 22879 | MON1B | 151 | 0.735 | -0.0329 | No |
| 3 | 7248 | TSC1 | 158 | 0.718 | -0.0067 | No |
| 4 | 55275 | VPS53 | 278 | 0.609 | -0.0676 | No |
| 5 | 1601 | DAB2 | 327 | 0.577 | -0.0781 | No |
| 6 | 23216 | TBC1D1 | 355 | 0.563 | -0.0737 | No |
| 7 | 1080 | CFTR | 366 | 0.557 | -0.0573 | No |
| 8 | 10618 | TGOLN2 | 383 | 0.547 | -0.0456 | No |
| 9 | 56894 | AGPAT3 | 414 | 0.527 | -0.0451 | No |
| 10 | 801 | CALM1 | 424 | 0.518 | -0.0295 | No |
| 11 | 22796 | COG2 | 445 | 0.507 | -0.0225 | No |
| 12 | 7109 | TRAPPC10 | 475 | 0.488 | -0.0228 | No |
| 13 | 51112 | TRAPPC12 | 504 | 0.471 | -0.0232 | No |
| 14 | 57679 | ALS2 | 656 | 0.399 | -0.1164 | No |
| 15 | 5898 | RALA | 813 | 0.309 | -0.2172 | No |
| 16 | 7037 | TFRC | 824 | 0.303 | -0.2116 | No |
| 17 | 3312 | HSPA8 | 848 | 0.244 | -0.2180 | No |
| 18 | 27131 | SNX5 | 907 | -0.333 | -0.2461 | No |
| 19 | 3949 | LDLR | 929 | -0.353 | -0.2464 | No |
| 20 | 157 | ADRBK2 | 947 | -0.367 | -0.2431 | No |
| 21 | 7277 | TUBA4A | 997 | -0.404 | -0.2617 | No |
| 22 | 23046 | KIF21B | 1034 | -0.433 | -0.2695 | Yes |
| 23 | 966 | CD59 | 1040 | -0.436 | -0.2545 | Yes |
| 24 | 81631 | MAP1LC3B | 1060 | -0.452 | -0.2491 | Yes |
| 25 | 339122 | RAB43 | 1083 | -0.462 | -0.2454 | Yes |
| 26 | 1604 | CD55 | 1090 | -0.468 | -0.2299 | Yes |
| 27 | 5565 | PRKAB2 | 1094 | -0.474 | -0.2118 | Yes |
| 28 | 6709 | SPTAN1 | 1114 | -0.487 | -0.2049 | Yes |
| 29 | 2706 | GJB2 | 1115 | -0.490 | -0.1840 | Yes |
| 30 | 1282 | COL4A1 | 1165 | -0.534 | -0.1970 | Yes |
| 31 | 83548 | COG3 | 1211 | -0.588 | -0.2047 | Yes |
| 32 | 22931 | RAB18 | 1231 | -0.608 | -0.1927 | Yes |
| 33 | 8408 | ULK1 | 1243 | -0.622 | -0.1741 | Yes |
| 34 | 26130 | GAPVD1 | 1247 | -0.625 | -0.1497 | Yes |
| 35 | 10802 | SEC24A | 1251 | -0.639 | -0.1246 | Yes |
| 36 | 1956 | EGFR | 1266 | -0.657 | -0.1067 | Yes |
| 37 | 286 | ANK1 | 1284 | -0.672 | -0.0905 | Yes |
| 38 | 57533 | TBC1D14 | 1304 | -0.708 | -0.0741 | Yes |
| 39 | 1839 | HBEGF | 1328 | -0.751 | -0.0589 | Yes |
| 40 | 23431 | AP4E1 | 1359 | -0.827 | -0.0455 | Yes |
| 41 | 6711 | SPTBN1 | 1381 | -0.925 | -0.0213 | Yes |
| 42 | 8867 | SYNJ1 | 1387 | -0.979 | 0.0168 | Yes |
Table: GSEA details [plain text format]

  

Fig 2: REACTOME\_VESICLE\_MEDIATED\_TRANSPORT      
 Blue-Pink O' Gram in the Space of the Analyzed GeneSet

  

Fig 3: REACTOME\_VESICLE\_MEDIATED\_TRANSPORT: Random ES distribution      
 Gene set null distribution of ES for **REACTOME\_VESICLE\_MEDIATED\_TRANSPORT**
